# Home care practice behavior and its influencing factors of primary care providers: a multicenter cross-sectional study in Sichuan Province, China

**DOI:** 10.1186/s12912-024-01948-3

**Published:** 2024-05-02

**Authors:** Luling Zhou, Suzhen Liu, Hang Li

**Affiliations:** 1 West China Hospital, Sichuan University/West China School of Nursing, Sichuan University, Chengdu, China; 2 Department of Urology, West China Hospital, Sichuan University, Chengdu, China

**Keywords:** Primary care providers, Home care, Practice behavior, Cross-sectional, Influence factors

## Abstract

**Background:**

Primary care providers play an important role in home health care, and their practice behavior is significant for care quality and patient outcomes. This study aimed to assess the home care practice behavior of Chinese primary care providers and to explore the factors associated with the practice behavior.

**Methods:**

A multicenter cross-sectional design with a convenience sample was used to survey 863 registered primary care providers from 62 primary health care settings in Sichuan Province, China. Descriptive statistics, t-test or ANOVA for one-way analysis, and Pearson’s correlation analyses were used to compare the differences and examine the relationships between participants’ demographics and experience of home care services and practice behavior. Multiple linear regression models were performed to identify salient variables associated with the practice behavior from among demographic and home care experience.

**Results:**

The score of home care practice behavior questionnaire was 97.25 ± 21.05. The average scores for the dimensions of home visit preparation, assessment, medical care behavior and safety practice were 3.70 ± 0.95, 3.76 ± 1.02, 3.66 ± 1.03, and 3.20 ± 0.46, respectively. Home care practice behavior was associated with working years, working experience in general hospitals, work area, home care experience such as client types of home care, service frequency and willingness, explaining 21.5% of the total variance.

**Conclusion:**

Chinese primary care providers had a medium to high level of home care practice behavior but poor implementation of safety practice. The results may provide clues to increased focus and implementation of safety practice, as well as providing targeted measures based on influencing factors.

## Introduction

Populations around the world are rapidly aging because of falling fertility rates and increasing life expectancy [1]. According to the WHO, one in six people worldwide will be aged ≥ 60 years in 2030 [2]. Consistent with global trends, the National Bureau of Statistics of China reported that the proportion of people aged 60 and over was 18.70% in 2020, which is increasing year by year [3]. The one-child policy implemented over the years and economic pressures have led to increasing burdens on young people and decreasing the caregiving capacity of families [4, 5]. Meanwhile, home-based older adults often face a constellation of daily challenges, including multiple chronic conditions, functional impairment, frailty, and social and mental stressors [6], leading to increasing demand for healthcare in China [7–9]. Over the past two decades, there has been a gradual extension of health-care services from hospitals to the community and the home [10]. This shift has also been driven by the objectives of shortening the length of hospital stay, relieving medical resource constraints and holding the belief of ageing in place [11, 12].

Home care, which is also called home-based services, is a cost-effective approach for the treatment of stable medical conditions and the management of chronic conditions [13]. It provides individualized care to patients of any age and families in their living environment by health professionals, which encompasses a range of activities, from preventive health work to palliative care [14]. Home care can decrease care costs while simultaneously improving quality [15], reducing emergency department visits and avoidable hospitalizations [16], improving advance care planning [17], relieving pressure on hospital care and reducing the risk of cross-infection [18]. On the other hand, compared with hospital work, home care is still fraught with uncertainty due to factors such as service environment, service population and operational risks. Especially as shorter hospital stays result in more patients being discharged from the hospital more quickly, providing home care while they are still uncomfortable is becoming increasingly complex [19].

It is noteworthy that the results of existing reports on home care were not ideal. A Canadian study found an incidence rate of 13.2% for adverse events in home care, of which one-third were considered preventable [20]. A national study in Japan reported that only 26.5% of facilities did not report adverse events in home-care settings [21]. Other countries had also mentioned the frequent occurrence of inadequate home care [22, 23]. The quality and safety of home health care need to be emphasized.

In China, home care has a late start but requires a high level of service competence from medical staff. First, there are clear requirements on the years of professional experience of medical staff, including more than three years of work experience for doctors, five years for nurses and three years for rehabilitation therapists [24]. In addition to basic medical operation skills and professional knowledge, they also need to be good at identifying and solving problems, communication and collaboration, and risk prevention. Not only do they share these common characteristics, different healthcare professionals also have their own specialties and characteristics. For example, doctors can prescribe medication, nurses can perform wound dressing changes and care, and rehabilitators can provide rehabilitation. However, they are both client-centered and have the goal of solving problems for their clients.

Apart from the personnel requirement, home care is also improving and developing in various aspects, including the way clients access the services, who provides them, and how they are provided. At present, clients access services primarily through basic medical insurance and long-term care insurance to support this, although the insurance has conditions for payment and can only partially cover the costs [25]. Home care fully meets the client-centeredness. According to the client’s wishes and specific service needs, the hospital staff will confirm the service information with the client in advance and then select the corresponding professionals and instrumental materials to bring the client a good treatment experience. Most of the time, home care providers are mainly nurses, followed by doctors [26]. During the process, medical staff will always follow up with the client and keep track of their health conditions. Although medical staff come from different institutions and backgrounds, they can communicate with each other, and seek help from other specialists in higher-level hospitals if they encounter problems. In particular, Internet + Care as a form of service delivery is emerging, which facilitates home care practice behavior for medical professionals [7, 27].

Home care practice behavior has an important influence on the quality of home care. The core home care practice behavior is the ability to perform clinical nursing care that is based on the medical worker’s ethical thinking and accurate practice skills [28]. The American Nurses Association Scope and Standards of Home Health Nursing Practice reported six professional practice standards and ten professional performance standards to articulate essential practice behavior [29]. A study subsequently further describes the implementation of the ANA standards in the home setting, giving examples of specific practice behavior competencies [30]. Another literature reported 10 competencies needed for home health care services, with care assessment as the first element [31]. The content of these competencies also informs the standards for practice behavior.

The state is actively promoting the development of home care and gradually regulating practice behavior. In 2020, the National Health Care Commission issued the document “notice on strengthening home medical services for elderly individuals“ [32], which proposed increasing the supply of home care services for the elderly in one step and put forward specific requirements for service institutions and medical workers. Among the specifics are stipulating that primary health care institutions are one of their main providers, adopting practice behaviors of medical staff such as signing agreements with service clients, comprehensively assessing disease, psychological, social support, environmental condition, and safety precaution. To enhance the safety of home care, there are qualification checks before clients can access services, including their proof of identity, case information, and family contracting agreements [32]. In addition, medical staff providing services were needed to attend and pass training organized by the hospital before they can formally provide services [32]. The role of primary health care and primary staff in family disease management is increasingly prominent [33, 34].

Overall, home care services have attracted the attention of a growing number of scholars. But researches mostly focusing on patients’ needs, willingness, or service availability [4, 7, 35–37], are lacking in exploring the home care practice behavior of medical professionals in primary care settings. Moreover, there may be variations in what home care service entails across countries and regions due to differences in the organization and structure. The purpose of this study is to describe the current situation of primary care providers’ practice behavior while providing home care services and to explore the factors that influence it to provide a reference for subsequent improvement of practice behavior competence, enhancing service quality and promoting patient outcomes.

## Methods

### Design

A multicenter cross-sectional survey was conducted in Sichuan Province, China. Data were collected from August 2021 to June 2022.

### Study setting and sample

The study was conducted in primary health care settings in Sichuan, China, with 62 facilities (including community hospitals, community health service centers, township health centers and other primary institutions) in 7 prefectures participating in this study (the service functions of these institutions are all based on basic public health and basic medical services, with the difference being that community health service centers are community-based and family-based, with residents of urban communities as their main service recipients, while township health centers are rural-based, with township residents as their service recipients. Community hospitals, on the other hand, can only be declared for approval when the health centers’ beds, size, outpatient capacity and other requirements have reached a certain standard). The participants were primary care providers who met the following inclusion criteria: (1) had obtained a licence to practice and were officially employed; (2) had worked in a primary health care organization for more than 12 months; (3) had experience with home-based medical services; and (4) voluntary participation. The exclusion criterion was personnel on extended leave or who changed jobs and no longer provided direct medical care and other professional services. A total of 863 medical workers from primary care settings were invited to participate in this survey.

### Data collection

The survey was asked to include as many primary care providers as possible using a convenient sampling method. After obtaining the consent of the institution’s managers, a staff member of the institution was hired as a surveyor and trained accordingly on the purpose and requirements of the survey. An electronic questionnaire link was sent to the surveyor, who in turn sent it to the medical staff of the institution. The surveyor explained the purpose and significance of the survey with the medical staff, and after agreement, signed an electronic informed consent form before completing the questionnaire, which had the same instructions for filling out the form and set each IP address to be filled out only once.

### Measurements

Self-designed questionnaires were used in the present study.

#### Demographic information

The survey collected data about basic information of primary care providers such as age, gender, working years, whether they have part-time employment in other organizations, working experience in a general hospital, level of education, professional title, role of medical workers and work area.

#### Home care experience of primary care providers

The home care experience included the years of providing home care, clients of home care, the frequency of service provided by provides, and the willingness to serve.

#### Home care practice behavior

The home care practice behavior questionnaire was developed based on insights from previous findings on home health care guidelines and other literature [29, 31, 38–40], as well as the content of national documents on the provision of home care services [24, 32]. The content of the scale, which did not involve targeted terminology for different specialties, evaluated the overall practical behavioral situation and process of healthcare professionals in providing home care services. The questionnaire was also applicable to healthcare professionals from different backgrounds. The questionnaire consisted of 27 items and four dimensions: home visit preparation (5 items), assessment (10 items), medical care behavior (6 items) and safety practice (6 items). Every term used five levels of frequency, and the total score of the scale was 27–135 (items 25–27 were reverse scored). Higher scores indicated better execution of home care practice behavior by primary care providers. During development of the questionnaire, a total of seven experts were invited to conduct questionnaire evaluations, including one community nursing educator, one community manager, one home care rehabilitation physician specialist, two home care nursing specialists, and two home care medical specialists. The research team modified the questionnaire to determine the final version based on the recommendations of seven experts. A presurvey of 30 medical workers showed that they could understand the questionnaire content and easily make judgments. The internal consistency of the questionnaire was 0.946, as measured by Cronbach’s alpha. The scale-level content validity index (S-CVI) was 0.96, and the item-level content validity index (I-CVI) ranged from 0.71 to 1.00 using an expert panel. Exploratory factor analysis was performed using a sample of 200 cases, which included doctors, nurses, and other medical personnel. The KMO was 0.928, and Bartlett’s test of sphericity was significant (*p* < 0.01). Four common factors with eigenvalues greater than 1 were extracted. Among them, the eigenvalues ranged from 1.261 to 13.270, and the cumulative variance contribution was 72.741%.

### Ethical approval

This study and its design received approval from the Biomedical Ethics Committee of West China Hospital of Sichuan University (Approval No. 2020 − 165). Furthermore, the primary care providers were assured that the findings would only be used for research purposes.

### Data analysis

We conducted a preliminary check and cleanup of the collected data. Continuous data were expressed as the mean and standard deviation. Categorical data were expressed as percentages. The current status of home care practice behavior was calculated by adding up the scores of all individual measures, using a t-test or ANOVA for one-way analysis, and variables with two-tailed tests with a significance of *P* < 0.2 were entered into the linear regression equation. Finally, in the linear regression results, *P* < 0.05 was considered statistically significant for this test. We used SPSS, version 25.0, to analyse the data.

## Results

### Demographic characteristics

A descriptive analysis of the general demographic and work-related information of the participants is shown in Table 1. One-third of them were ≤ 30 years old. The majority (92.1%) were women. There were 324 workers (37.5%) who had worked for 5 years or less, 247 (28.6%) who had worked for 6–10 years, and 292 (33.9%) who had worked for more than 10 years. Only 16 individuals worked part-time at other medical institutions. A total of 48.7% of them had working experience in general hospitals. More than half of the staff (54.7%) had a bachelor’s degree, followed by a college degree (38.5%). The professional titles of the staff were mainly junior (48.4%) and middle level (29.8%). Among all employees, there were 261 doctors (30.3%), 473 nurses (54.8%) and 129 other personnel (14.9%) (including rehabilitation therapists, pharmacists and other workers). In addition, the majority of staff (73.6%) worked in urban areas, with only 26.4% working in districts, counties or townships outside urban areas.

**Table 1:** Demographic characteristics of the participants (*n* = 863)

Variable	*n* (%)	Variable	*n* (%)
Age (years)		The highest level of nursing education	
≤ 30	267(30.9)	Secondary education	51(5.9)
31–40	364(42.2)	College degree	332(38.5)
>40	232(26.9)	Bachelor’s degree	472(54.7)
Gender		Graduate degree	8(0.9)
Male	68(7.9)	Professional title	
Female	795(92.1)	Below junior title	81(9.4)
Working years		Junior title	418(48.4)
≤ 5 years	324(37.5)	Intermediate title	257(29.8)
6 to 10 years	247(28.6)	Senior title	107(12.4)
> 10 years	292(33.9)	Role of Medical workers	
Part-time employment in other organizations		Doctor	261(30.3)
Yes	16(1.9)	Nurse	473(54.8)
No	847(98.1)	Other personnel	129(14.9)
Working experience in general hospitals		Work area	
Yes	420(48.7)	Urban areas	635(73.6)
No	443(51.3)	Rural areas	228(26.4)

### Home care experience of primary care providers

The experience of home care by primary care providers is shown in Table 2. More than half of them (57.4%) had been providing the service for < 3 years. Current services were provided to a variety of clients, with the majority being older adults ≥ 60 years or older, followed by people with disabilities and maternity. A total of 43.8% provided services to ≥ 3 types of clients. Only approximately 1 in 5 provided home care ≥ 1–2 times per week. Of all employees, the majority said they would be willing to perform home care services.

**Table 2:** Home care experience of the participants (*n* = 863)

Variable	*n* (%)	Variable	*n* (%)
Years of providing home care		Frequency of home care service	
<3 years	495(57.4)	≥ 1–2 times per week	166(19.2)
3–5 years	180(20.8)	About once a month	231(26.8)
≥ 6 years	188(21.8)	About once every three months	179(20.7)
Clients of home care (multiselect)		Less than once every six months	287(33.3)
Children < 3 years old	267(30.9)	Willingness to serve	
Maternity	470(54.5)	Unwillingness	89(10.3)
Disabled person	527(61.1)	Not matter	104(12.1)
Patients discharged from hospital	237(27.5)	Willingness	490(56.8)
People ≥ 60 years old	665(77.1)	Very willing	180(20.8)
Client types of home care			
1 type	234(27.1)		
2 types	251(29.1)		
≥ 3 types	378(43.8)		

### Home care practice behavior

The score of the home care practice behavior questionnaire was 97.25 ± 21.05. The average scores for the dimensions of home visit preparation, assessment, medical care behavior and safety practice were 3.70 ± 0.95, 3.76 ± 1.02, 3.66 ± 1.03, and 3.20 ± 0.46, respectively. There was a significant positive correlation between all four dimensions in bivariate analyses. In particular, the safety practice score had the smallest correlation coefficient with the other three dimensions (*r* < 0.3, *P* < 0.001), and the correlation coefficients among the other dimensions were all > 0.7 (*P* < 0.001).

### Associated factors

The univariate analyses showed that the primary care providers who were aged 30 years or younger, female, worked 5 years or less, had part-time employment in other organizations, had working experience in general hospitals, worked in urban areas, provided home care ≥ 3 years, had over 3 types of service clients, worked ≥ 1–2 times per week, and were very willing to serve had better home care practice behavior (Table 3).

**Table 3:** Univariate analyses of the factors associated with home care practice behavior (*n* = 863)

Variable	home care practice behavior	t/F	*P*
Age (years)		3.896	0.021
≤ 30	100.13 ± 20.62		
31–40	96.20 ± 22.55		
>40	95.60 ± 18.75		
Gender		-1.443	0.149
Male	93.72 ± 23.74		
Female	97.56 ± 20.80		
Working years		9.919	<0.001
≤ 5 years	101.32 ± 20.11		
6 to 10 years	94.48 ± 21.65		
> 10 years	95.09 ± 20.93		
Part-time employment in other organizations		1.595	0.111
Yes	105.00 ± 18.39		
No	97.10 ± 21.08		
Working experience in general hospitals		2.688	0.007
Yes	99.23 ± 20.69		
No	95.39 ± 21.25		
The highest level of nursing education		1.049	0.370
Secondary education	95.71 ± 19.21		
College degree	96.73 ± 21.54		
Bachelor’s degree	97.59 ± 21.01		
Graduate degree	109.13 ± 8.97		
Professional title		0.926	0.429
Below junior title	99.31 ± 20.54		
Junior title	97.70 ± 22.21		
Intermediate title	95.59 ± 20.92		
Senior title	97.96 ± 16.62		
Role of medical workers		0.333	0.717
Doctor	97.27 ± 18.99		
Nurse	97.63 ± 21.82		
Other personnel	95.84 ± 22.21		
Work area		1.789	0.074
Urban areas	98.02 ± 20.20		
Rural areas	95.12 ± 23.19		
Years of providing home care		2.644	0.072
<3 years	95.89 ± 23.07		
3–5 years	99.02 ± 17.62		
≥ 6 years	99.17 ± 18.13		
Client types of home care		17.775	<0.001
1 type	92.68 ± 23.19		
2 types	94.41 ± 18.82		
≥ 3 types	101.98 ± 20.13		
Frequency of home care service		16.618	<0.001
≥ 1–2 times per week	103.98 ± 18.69		
About once a month	100.98 ± 17.57		
About once every three months	95.97 ± 20.87		
Less than once every six months	91.17 ± 23.22		
Willingness to serve		59.480	<0.001
Unwillingness	86.54 ± 20.40		
Not matter	83.97 ± 20.57		
Willingness	96.75 ± 19.30		
Very willing	111.59 ± 17.35		

Regression analyses examining covariates of home care practice behavior are presented in Table 4. In this model, working years, working experience in general hospitals, work area, experience of home care services such as client types, home care frequency and willingness were significant correlates, explaining 21.5% of the total model variance.

**Table 4:** Regression analysis examining covariates of home care practice behavior

Model	B	SE	Beta	T	*P*
(constant)	81.885	5.622		14.565	<0.001
Age (years)					
31–40	-1.200	1.655	-0.028	-0.725	0.469
>40	0.143	2.074	0.003	0.069	0.945
≤ 30	---				
Gender(female)	1.241	2.429	0.016	0.511	0.610
Working years					
6 to 10 years	-4.641	1.689	-0.100	-2.748	0.006
> 10 years	-3.463	1.878	-0.078	-1.844	0.066
≤ 5 years	---				
Part-time employment	4.497	4.775	0.029	0.942	0.347
Worked in general hospitals before	3.102	1.324	0.074	2.343	0.019
Working in urban areas	3.202	1.491	0.067	2.147	0.032
Years of providing home care					
3–5 years	2.924	1.693	0.056	1.727	0.084
≥ 6 years	3.469	1.797	0.068	1.930	0.054
<3 years	---				
Client types of home care					
1 type	-4.274	1.618	-0.090	-2.642	0.008
2 types	-3.210	1.567	-0.069	-2.048	0.041
≥ 3 types	---				
Frequency of home care service					
≥ 1–2 times per week	8.047	1.900	0.151	4.235	<0.001
About once a month	5.729	1705	0.121	3.360	0.001
About once every three months	3.636	1.880	0.070	1.934	0.053
Less than once every six months	---				
Willingness to serve					
Not matter	-1.932	2.714	-0.030	-0.712	0.477
Willingness	8.257	2.185	0.194	3.778	<0.001
Very willing	21.285	2.513	0.411	8.471	<0.001
Unwillingness	---				

*F* = 14.133, *P* < 0.001, *R*^*2*^ = 0.232, Adjusted *R*^*2*^ = 0.215

## Discussion

### Key results and interpretation

The purpose of this study was to examine how health care workers provided home care services in Chinese primary care settings. Among the 863 health care providers, 57.4% had been providing home care for < 3 years, and more than half provided the services once every 3 months or less than once every 6 months. These results indicated that the workload of home care in the primary care setting was lighter. This may be related to the late start of home care in China. Many organizations are limited in the number of service programs they can provide to their clients. While the main providers were still nurses [26], the frequency of services provided by other medical personnel such as rehabilitators and dietitians was relatively low in this study. Another important reason was that the insurance reimbursement measures for home health care were not yet perfect [41]. The number of services and insurance options that can be reimbursed for home health care were limited, and rarely reimbursed 100%. Many services needed clients to pay out-of-pocket, which also led to a low supply of services even though there was demand from clients. This needed the state to expand coverage in accordance with national conditions and to include more programs for home health care in the reimbursement. It could be tried that a family shares an account and pays through the young worker’s health insurance. Insurance companies can also develop commercial health insurance that covers home care to increase reimbursement pathways [42].

In recent years, the state had introduced several policies to promote the development of home care [24, 32, 43]. Especially after entering an aging society and implementing the two-child policy, Chinese residents had an increasing demand for health care and health promotion. More emphasis had been placed on providing services for elderly individuals, disabled and other special populations. This survey showed that the people receiving services were mainly elderly individuals ≥ 60 years old, disabled individuals and pregnant women, indicating that primary medical institutions were implementing national policies in place. In addition, the majority of primary care providers were willing to provide home care, closer to the results of a nationwide survey conducted in China [7].

Overall, the home care practice behavior of primary care providers was in the middle to upper level (97.25 ± 21.05). The best was the assessment of the clients, and the worst was the safety practice, which included patient and medical staff safety. Specifically, this referred to the adverse events that affect patient safety and unforeseen circumstances that affect the health and safety of medical staff themselves during the course of providing services in the home. A previous study conducted in Hunan Province, China, showed that nurses were concerned about the safety of patients and their own practice during home visits [44]. Furthermore, some relevant studies had also reported the need to enhance the safety management of home care [45, 46]. The six items in the safety practice dimension of this study included risk identification, use of the APP locator system, wearing an alarm device, occurrence of patient accidents, medical staff safety accidents, and requesting support, with the lowest scores for the items using the APP locator system and wearing an alarm device, which was in line with the results of other researchers [47]. Primary care organizations may have insufficient hardware facilities (including alarm devices) due to limited funds and cumbersome equipment purchase processes [47]. The lowest safety scores may also be related to factors such as smaller service jurisdictions, relative familiarity with clients in the area, and low attention from medical staff [35]. The attention of medical institutions should be drawn to how to help them guarantee the use of material resources, raise the medical staff awareness of safety and protection, and thus regulate their safety practice behavior. The other two dimensions with scores in the middle were home visit preparation and medical care behavior. This included taking an itemized inventory of item preparation prior to the visit and strict adherence to specifications after the visit. Adequate preparation and proper implementation of these two elements also mean double security and better service outcomes for both doctors and patients, and vice versa.

Our study found that home care practice behavior was worse for those who had worked ≥ 6 years. This result may differ from normal thinking. It may be that home health care in China started late and had only been gradually standardized in the last five years [32]. Most of the medical staff who had worked for ≥ 6 years were already at an intermediate level in primary health institutions and had more opportunities to take on managerial responsibilities than to provide services in the home. Lower seniority and relatively younger staff may undertake more home service work. Despite the shorter working time, the young medical staff in this survey had relatively higher qualifications and stronger basic competencies in their own right. In addition, those who had been working for more than six years usually faced stress, which comes from marriage and childbirth, restricted job development, and family care burden [48], and increased as age and working years increased, making them more susceptible to burnout and affecting the completion and quality of home care [49, 50]. Hence, focusing on people who work longer and understanding their needs is essential to enhancing their practice behaviors.

Previous worked in a general hospital and current working in an urban area were contributing factors to the home care practice behavior of primary care providers. Personnel who had worked in general hospitals had better practice behavior. They usually had extensive experience in the treatment of complex diseases and emergency management. Not only were there many opportunities for training and learning, but there was also a stricter code of ethics and behavior. In particular, medical personnel have been needed to undergo 2–3 years of standardized training in general hospitals after graduating from school in recent years [51], rotating through different types of departments to acquire comprehensive theoretical and operational skills, better and rapidly adapt to the clinical environment and enter the medical role [52]. The level of medical staff behavior varied in different hospitals, as well as in different regions. Residents living in rural areas had lower utilization rates of outpatient clinics and preventive health services, and thus less access to services than those in urban areas [53, 54]. At the same time, the lower economic income and poorer environment in rural areas led to a concentration of professionals mostly in urban areas, further contributing to the imbalance in human resources, which was the reason for the better home care practice behavior of medical personnel in urban areas. The state is currently reducing disparities by taking measures such as the sinking of high-quality medical resources from cities and alliances of urban and rural institutions, but the specific implementation measures and their effects in the future have yet to be further explored.

Our research showed that the home care experience was related to their practice behavior. Greater types of clients served and higher service frequency were important factors for better home care practice behavior. Home care is faced with numerous uncertainties, such as unfamiliar environments, complex disease and care needs, and diverse work content [55, 56]. This placed higher demands on the behavior of medical personnel. Having a thorough understanding of the client’s health condition and preparing the relevant materials and documents according to their needs was essential prior to the visit. Immediately after the visit, the client was assessed and treated accordingly. During this process, various unexpected situations may occur, and medical staff need to react quickly and master the emergency treatment process. The greater the variety of clients served and the higher frequency of service, the more conducive it was to the enhancement of the professional skills and emergency response capabilities of the medical staff. This experience-based behavior enabled primary care providers to assess what a situation requires and act accordingly based on both professional and conditional practical experience [57, 58]. Primary health care institutions can organize regular training and assessment related to the operational skills and theories needed for common service items of home health care. At the same time, drills are conducted for common emergency resuscitation and adverse events. The effectiveness of the training can be enhanced by making full use of modern Internet technology, simulation laboratories and VR [59, 60].

As revealed in the multiple linear regression models, willingness to serve was significantly associated with home care practice behavior. The higher home care willingness of providers, the more motivated they were to work and the more able they were to engage in self-directed learning and regulate their own behavior. In this study, we also found that 77.6% of primary care providers expressed that they would be willing to provide home care for clients, which was in the middle of the range compared to the results of previous studies [7, 61]. According to the previous literature [7, 62–64], willingness to engage in home care may be influenced by a variety of factors, such as age, education, income, and practice safety. Medical professionals with low willingness had lower quality of care and poorer job stability, similar to the poorer home care practice behavior findings in this study. According to the hospital’s own situation, managers can strengthen publicity to deepen providers’ understanding of home care. On the other hand, they should understand the needs of workers and take targeted measures such as providing safety support and adjusting welfare benefits to increase their willingness to provide home care services, thus promoting the standardization of home care practice behavior.

### Limitations

Because of the cross-sectional design, we could not judge the combined effect of multiple factors on home care practice behavior, nor could we infer causality. Although this study surveyed primary care providers at 62 medical institutions, they were all located within Sichuan Province and were not representative of national data. In addition, the survey instrument used in this study was tested by exploratory factor analysis. And other methods, such as confirmatory factor analysis, can be used to improve the reliability and usefulness of the questionnaire in subsequent studies. We also encountered some difficulties during the study, including irregularities in questionnaire completion, insufficient sample size, and low participation of certain organizations. In the future, we will strengthen the publicity of home health care and emphasize to the participants the great significance of questionnaire completion for the study to include a larger number of organizations and populations.

## Conclusions

The home care practice behavior of primary care providers scored above the questionnaire average and was in the middle to upper level. The assessment was the best performing, followed by home visit preparation and medical care behavior. The safety practice did the worst, which needs to be focused on and further improved. Factors associated with home care practice behavior included working year, working experience in a general hospital, work area, home care experience and willingness to serve. Moreover, such innovative programs should be considered within the Chinese primary care provider context to support this crucial patient care workforce.

## Data Availability

The datasets used and analysed during this study are available from the corresponding author upon reasonable request.
